# Supplementary material for: Paraffin-enabled graphene transfer
Source: Nat Commun. 2019 Feb 20;10:867. doi: 10.1038/s41467-019-08813-x (PMC6382797; doi:10.1038/s41467-019-08813-x)
Supplement: Supplementary file 1 — Supplementary Information [file 41467_2019_8813_MOESM1_ESM.pdf]

Supplementary Information for

Paraffin Enabled Graphene Transfer

Leong *et al.*

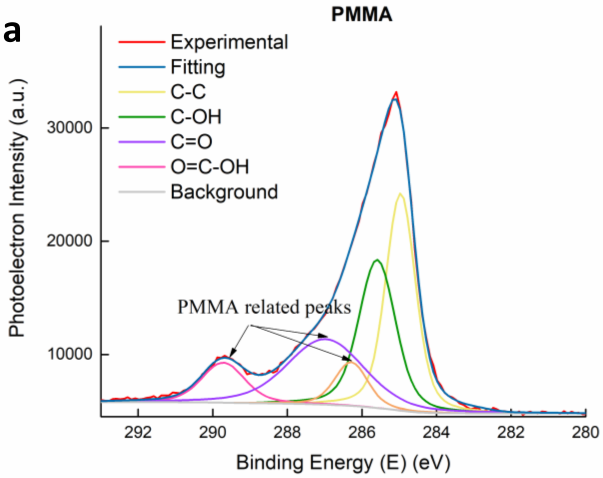

Supplementary Fig. 1 | X-ray photoelectron spectroscopy (XPS) spectrum of **a,** PMMA- and **b,** paraffin-supported graphene transferred on Si/SiO_2 substrate. Arrows in **a** indicate the peaks related to PMMA residual.

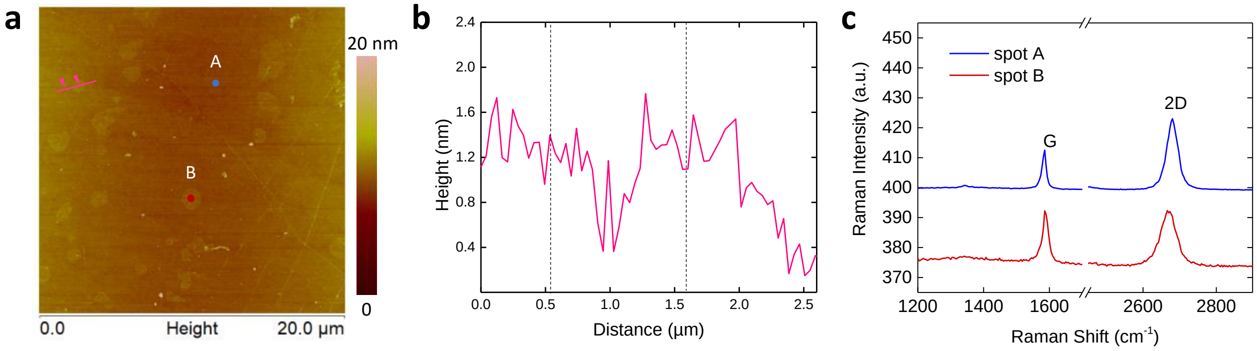

Supplementary Fig. 2 | a, Typical AFM topography image of a paraffin-transferred graphene on a Si/SiO_2 substrate. **b,** Height profile along the line marked. **c**, Raman spectra of the two spots marked. As can be seen, our paraffin-transferred graphene has a clean and smooth surface such that 1-µm-sized bilayer graphene domains can be clearly observed under AFM and confirmed by Raman analysis.

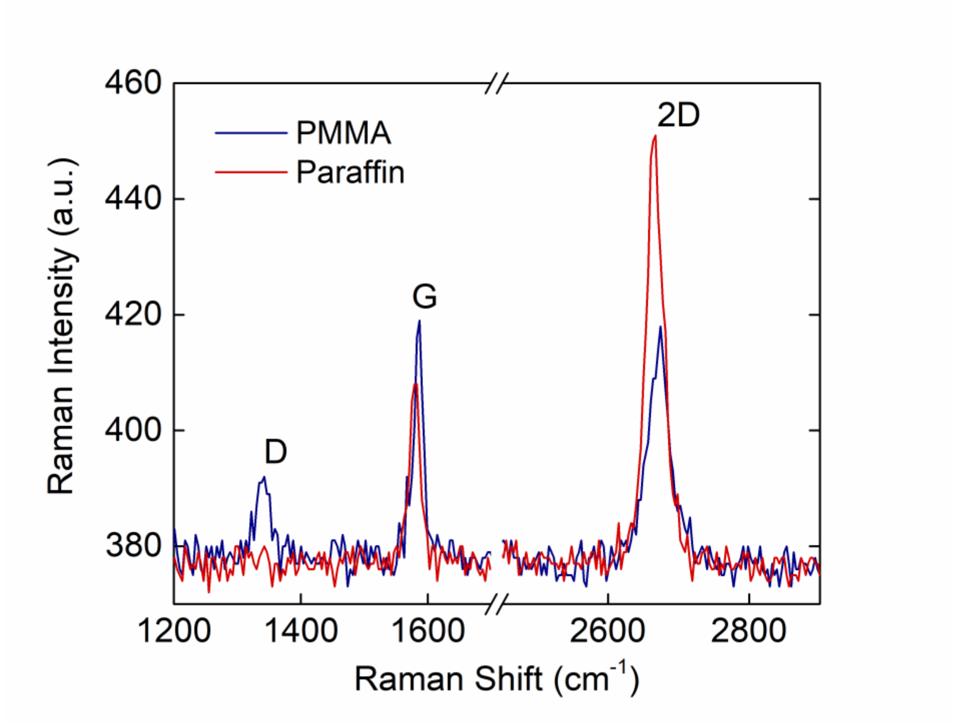

Supplementary Fig. 3 | Typical Raman spectrum of the PMMA- and paraffin-transferred graphene on Si/SiO_2 substrate.

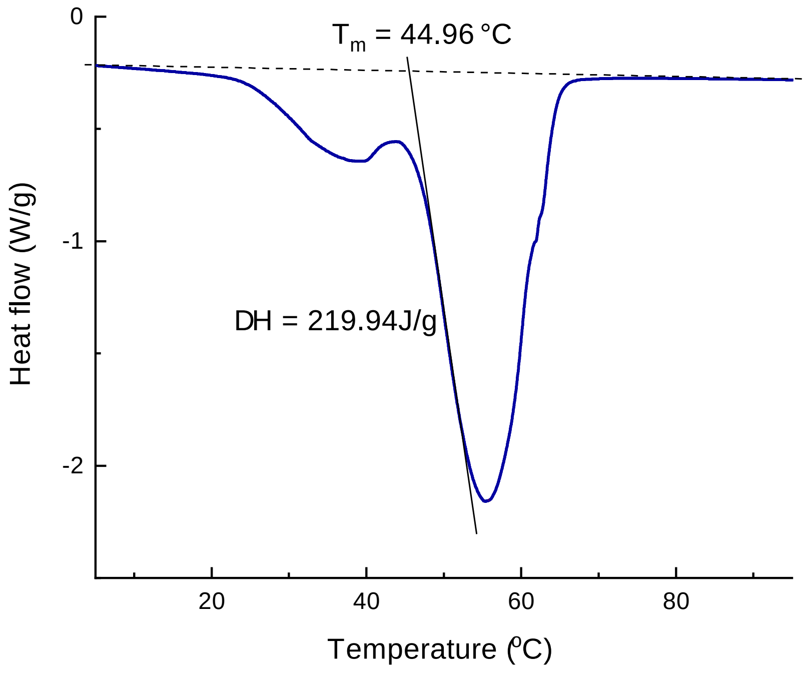

Supplementary Fig. 4 | Differential scanning calorimetry measurement of the paraffin used in this work.

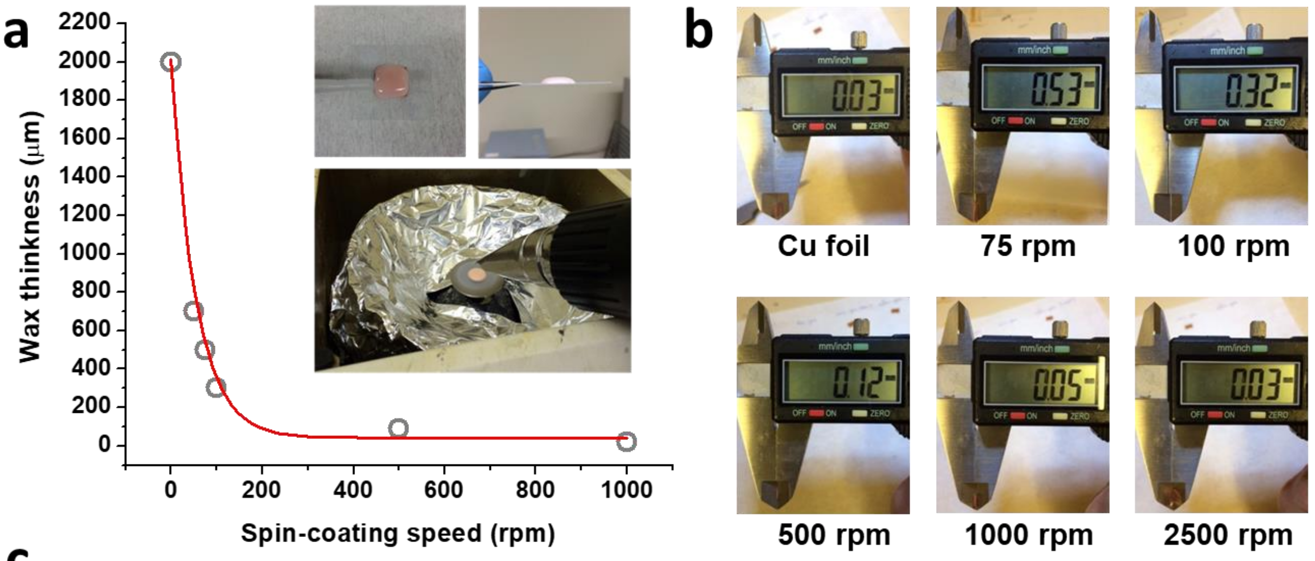

c

Spin-coating speed (rpm)	0	50	75	100	500	1000	2500
Wax thickness (μm)	2000	700	500	290	90	20	< 10 μm

Supplementary Fig. 5 | a, The paraffin wax thickness as a function of spin-coating speed. Insets: Top and side views of paraffin-coated CVD graphene on Cu foil. Heat gun is used to ensure the paraffin wax is always in liquid state prior to and during the spin-coating process. **b,** A digital calliper is used to measure thickness of all wax-coated graphene on a 32 μm thick Cu foil. **c,** Table of results showing the thickness of paraffin wax when the speed of spin-coating changes.

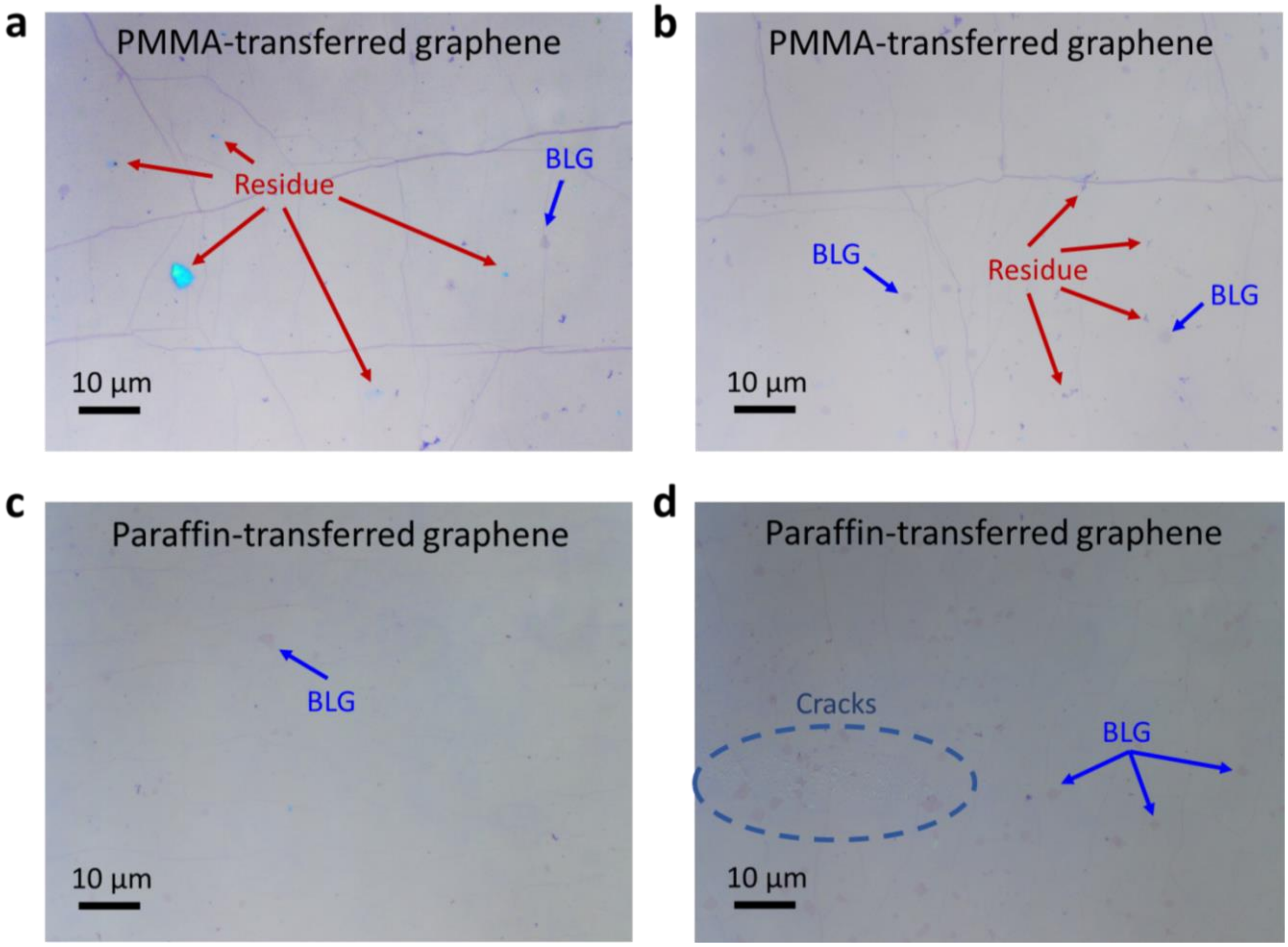

Supplementary Fig. 6 | Comparison of PMMA- and paraffin-transferred graphene. **a-b,** Typical optical images of PMMA-transferred graphene on a Si/SiO_2 substrate. **c-d,** Typical optical images of paraffin-transferred graphene on a Si/SiO_2 substrate. Blue arrows indicate some of the bilayer graphene (BLG) domains. Red arrows indicate the presence of polymer residues. Dotted circles indicate minor cracks randomly generated in the graphene due to the incomplete removal of residual water.

Supplementary Note 1: Modified PMMA-supported graphene transfer process

Here, we conducted a modified PMMA-supported graphene transfer with minimal process variants to that of paraffin, to confirm the advantageous of paraffin over PMMA as a sacrificial support layer. First, a layer of PMMA 950 A5 (Microchem Inc.) was spun at 2,500 rpm for 1 min on the graphene synthesized above Cu foil. The PMMA-coated graphene was then baked in an oven at 80 °C for 1 h. Next, the sample was floated on top of Cu etchant (Copper Etchant TFB, Transense) for 30 min to remove the growth substrate. The PMMA-supported graphene film was then rinsed with deionized water multiple times. Subsequently, the sample was transferred onto 40 °C deionized water surface and kept at the same temperature for at least 1 h. The destination substrate (*i.e.* Si/SiO_2 substrate) was then used to scoop the PMMA-supported graphene sample from the 40 °C deionized water from one end. After that, the sample was baked at 40 °C in an oven for more than 24 h. Subsequently, the sample was soaked in a mixture of hexane and acetone (1:1) at room temperature for 12 h to remove the PMMA support layer. The sample was then rinsed with IPA followed by nitrogen blow dry. Supplementary Fig. 7c shows a typical atomic force microscopy (AFM) height profile image of CVD graphene transferred on Si/SiO_2 substrate using the modified PMMA-supported graphene transfer. For direct visual comparison, we reprinted the AFM height profile image of the graphene transferred with conventional PMMA support layer from Fig. 4a in Supplementary Fig. 7a below. As can be seen, the AFM height profile images of graphene transferred with either modified or conventional PMMA-assisted transfer approaches looked comparable in terms of wrinkle density and polymer contamination.

For comparison, we also conducted a modified paraffin-supported graphene transfer experiment where all process parameters were remained the same as the paraffin-supported graphene transfer process described in the Methods (see main text), except for the last sacrificial layer removal step. In this specific experiment, the paraffin support layer was removed using a mixture of hexane and acetone (1:1) at room temperature for 12 h, rather than pure hexane, leaving monolayer graphene on the destination substrate. A typical AFM height profile image of the CVD graphene transferred on Si/SiO_2 substrate using the

modified paraffin-supported transfer approach is shown in Supplementary Fig. 7d. As can be clearly seen, the graphene transferred with either standard or modified paraffin support layer shows substantial reduction in terms of wrinkles and polymer contamination.

Overall, the results verify the benefits of paraffin over PMMA, as a sacrificial support layer for graphene transfer technology, in terms of contamination and wrinkle reduction.

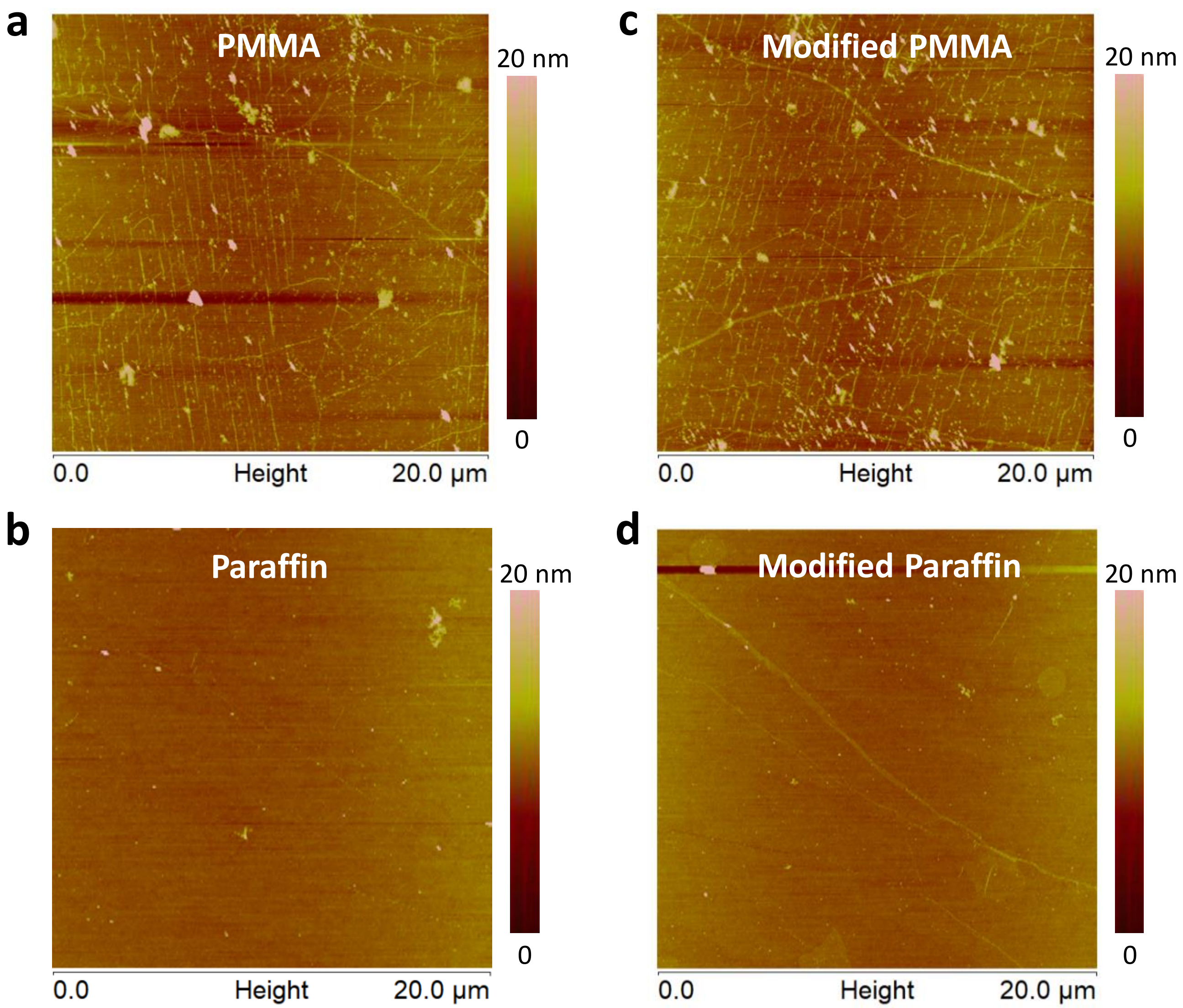

Supplementary Fig. 7 | Typical AFM height profile images of graphene film prepared with **a,** PMMA, **b**, paraffin, **c,** modified PMMA, and **d,** modified paraffin support layers transfer method, respectively. Supplementary Fig, 7a, b was reprinted from Fig. 4a, b, for direct visual comparison.

Supplementary Note 2: Descriptions of DFT calculations

Since PMMA and paraffin may form radicals due to thermal degradation at elevated temperatures: PMMA may form tertiary radicals (most stable) while paraffin may form primary radicals that are much less stable than tertiary radicals. Therefore, PMMA radicals are very likely to be sufficiently stable to eventually react with graphene forming covalent bonds, especially in the presence of common defects such as vacancies, whereas paraffin radicals are unlikely to exist for any significant period of time.
